# 肺鳞癌与肺腺癌的免疫组化指标的诊断及预后意义

**DOI:** 10.3779/j.issn.1009-3419.2014.06.13

**Published:** 2014-06-20

**Authors:** 云帆 马, 梦颖 范, 克能 陈

**Affiliations:** 100142 北京，北京大学肿瘤医院暨恶性肿瘤发病机制及转化研究教育部重点实验室胸外一科 Key Laboratory of Carcinogenesis and Translational Research (Ministry of Education), Department I of Thoracic Surgery, Peking University Cancer Hospital, Beijing 100142, China

肺癌已成为全世界范围内的一个重大公共卫生问题，在中国，虽然肺癌已是死亡率与发病率最高的恶性肿瘤，但其发病率仍未达到最高峰^[1]^。随着肺癌治疗手段的改变，近年来疗效有所改善，然而其预后普遍较差，5年生存率约为15%。非小细胞肺癌（non-small cell lung cancer, NSCLC）占全部肺癌的80%以上，其中大多数组织学类型为腺癌（adenocarcinoma, ADC）（40%-50%）和鳞状细胞癌（squamous cell carcinoma, SQCC）（30%）。NSCLC的治疗原则长期依据以解剖学为基础的TNM分期。随着对肺癌基因背景的认识，更多的理论加入了肺癌治疗选择的依据，例如：抗叶酸化疗药物“培美曲塞”只对肺ADC有效^[2]^，贝伐单抗则应归避用于肺SQCC^[2]^，表皮生长因子受体酪氨酸激酶抑制剂（epidermal growth factor receptor tyrosine-kinase inhibitor, EGFR-TKI）治疗只对有*EGFR*基因突变者有效^[3]^，克唑替尼只对*ALK*基因重排的人群有效^[4]^。因此，肺癌的治疗策略已经从传统的以分期为基础的治疗模式转变为以组织学类型和基因突变为指导的个体化、精准的多学科治疗模式。随着NSCLC的研究进展，更多地依据临床、影像、病理生长类型、基因表达和分子生物学标志物等特征将NSCLC分成预后不同的多种亚型，从而指导临床治疗决策。

镜下形态特征一直是WHO肺癌分类的金标准。一般来讲，苏木精-伊红染色法（hematoxylin-eosin staining, HE）染色已足够区别肺ADC与非ADC。但在少数情况下，如穿刺获得的小标本，甚至细胞学标本，难以了解肿瘤全貌，Ⅲ期、Ⅳ期NSCLC患者由于不能手术切除肿瘤，小标本往往又是诊断唯一可利用的组织。或当肿瘤分化较差时，缺少肺ADC和SQCC的细胞学结构特征，分型就比较困难。因此出现了组织学类型不明确型非小细胞肺癌（non-small cell lung carcinoma-not otherwise specified, NSCLC-NOS）的概念^[5]^。2009年的一项研究^[6]^表明在活检标本中NSCLC-NOS的比例约为25%，而在细胞学标本中其比例达到40%。由于肺癌精确分型的需要，过去病理学单纯依靠形态学进行诊断的方式已受到了挑战。基于以上这些情况，免疫组化（immunohistochemistry, IHC）检测在NSCLC的亚分型中就显得十分重要。IHC因其简便、相对廉价和可靠的特点，已经得到了广泛的公认。IHC不仅可以对各种低分化肺癌进行分型，更适合于取材受限的小标本。2011年国际肺癌研究协会、美国胸科学会及欧洲呼吸学会（IASLC/ATS/ERS）公布了肺ADC的多学科分类原则，指出肺癌的亚分型不能单纯依靠传统的HE染色切片，特别是在诊断小标本和细胞学组织时，还需要IHC的指导^[5]^。虽然最佳的诊断标准还没有确立，但研究^[7]^已表明IHC提高了诊断的准确性和可重复性，减少了NSCLC小标本不能被亚分型的几率。

常用的鉴别SQCC指标为p63、p40和细胞角蛋白5/6（cytokeratin 5/6, CK5/6）。常用的鉴别ADC指标为甲状腺转录因子-1（thyroid transcription factor -1, TTF-1）、新天冬氨酸蛋白酶A（novel aspartic proteinase A, Napsin A）和细胞角蛋白7（cytokeratin 7, CK7）。本文就常用的IHC标志物的诊断意义及预后意义作一综述。

## 1 肺鳞癌IHC标志物的诊断及预后意义

### 1.1 肺鳞癌IHC标志物的诊断价值

#### 1.1.1 p63、p40在诊断肺SQCC中的应用

*p63*基因位于染色体3q27-29，与p53有结构同源性。p63有两个启动子，通过可变剪接产生两个相对的类蛋白：全长蛋白TAp63(含有反式激活域）和截短蛋白ΔNp63（缺乏反式激活域），即p40。TAp63有与p53类似的反式激活TA结构域，调控生长抑制基因的表达。ΔNp63同种型包含有一种替代转录惰性的“ΔN”域，拮抗TAp63和p53的活性。所以p63包含2个功能相反的亚型：TAp63-类p53肿瘤抑癌基因和ΔNp63-致癌基因。4A4抗体既识别TAp63，又可以识别ΔNp63，而p40抗体只识别ΔNp63。p63和p40均在细胞核染色。

研究^[8]^已经表明p63蛋白诊断肺SQCC的灵敏度接近100%。同时，p63在SQCC中稳定的表达阳性，与分化程度无关，是一个高度稳定的标志物^[9]^。p63的应用主要限制是它的特异性低，为60%-86%^[10]^。p63的在ADC中的阳性率约16%-65%^[11]^。在大多数的p63阳性的ADC中，表达是局灶的，但在个别病例接近典型的SQCC程度^[12]^，往往可能是ADC的实性变异。p63蛋白作为“鳞状标记物”的另一个重要限制是它可以表达在多种其它类型的肿瘤，特别是淋巴瘤，已报道其阳性反应达50%^[13]^。这可能造成误诊误治，因为大细胞淋巴瘤可表现为孤立性胸腔肿块，其上皮的形态也可以类似NSCLC。在这种情况下，p63表达强阳性可能将淋巴瘤误诊为SQCC。但总体来说，p63还是一个非常灵敏、特异的反映鳞状分化的标志物。

虽然ΔNp63（p40）和TAp63剪接变体都在NSCLC表达，但ΔNp63是更主要的异构体，它选择性地表达在SQCC中。2000年Hibi等^[14]^采用IHC第一个研究了p40抗体，根据23例肺癌分析发现p40对SQCC是完全的敏感和特异的，当时这一发现未被重视。直到Pelosi等^[15]^研究了20例肺SQCC全组织切片，随后又增加了46例小标本和细胞学标本，及手术切除的NSCLC标本，发现p40抗体不像p63的4A4抗体，对SQCC有100%的特异性。另一项研究^[16]^中，总共对150例肺ADC和50例肺SQCC采用p63（4A4）和ΔNp63（p40）进行免疫染色，所有SQCC中p63和p40均弥漫阳性，ADC中27例（18%）p63呈阳性反应，而p40均阴性。p40检测SQCC的敏感性相当于p63，但特异性却高于p63。

#### 1.1.2 CK5/6在诊断肺SQCC中的应用

CK是由分子量和等电点不同的20个多肽构成的一个大家族，CK分为两种类型，1型（CK9-20）是较小酸性多肽、2型（CK1-8）是较大的中性多肽。CK是一种中间丝蛋白，中间丝（7-11纳米）是真核细胞中主要的细胞骨架蛋白。CK家族都表达于上皮细胞，是反应上皮细胞分化有用的标志物。CK在细胞膜和细胞质染色。CK在维持正常上皮细胞恶性转化中发挥作用。研究^[17]^认为CK结构重排触发了染色质的重组，并提供了具有生长活性的转化细胞，在致癌机制中发挥直接作用。研究表明，CK的表达增加可能反映了肿瘤发生和发展过程中组织细胞结构的变化。

CK5/6属于中等大小的碱性角蛋白。在正常组织中，CK5/6主要表达于角化（上皮）和非角化（粘膜）的鳞状上皮，以及前列腺、乳腺和唾液腺的基底肌上皮细胞层。CK5/6也出现在上皮、粘膜鳞状上皮和肌上皮起源的良性和恶性肿瘤，如乳腺癌的基底层和间皮瘤。CK5/6是敏感的反映鳞状分化的标志物，报道中其诊断肺SQCC的敏感度为75%-100%^[18]^，特异性在Whithaus等^[10]^的研究中也达到96%。CK5/6在原发性肺ADC中表达阳性的比例很小（2%-8%）^[18, 19]^。

除了p63、p40和CK5/6外，还有许多其它的鳞状上皮标记物，包括34bE12、Desmocollin-3、S100A2、S100A7、SOX2、Glypican 3、miR-205，已经应用于从ADC中鉴别肺SQCC。这些标记物没有能与p40相匹配的灵敏性和特异性。唯一特异性接近100%的是Desmocollin-3，然而有报道其敏感性从52%-100%不等^[11]^。

### 1.2 肺鳞癌IHC标志物的预后价值

目前p63蛋白表达对NSCLC的预后的影响已有报道，但结果相互矛盾^[20, 21]^。在Renouf^[21]^和Au^[22]^的研究中，单因素分析p63高表达是患者良好预后的指标，但多因素分析没有意义。虽然目前p63在预测患者预后方面并没有明显的优势，但NSCLC患者*p63*基因3q扩增和p63过表达可以延长生存^[23]^。Ko等^[24]^的研究发现，在淋巴结阴性的Ⅰ期-Ⅱ期NSCLC患者中，RASSF1A甲基化和p63蛋白阴性表达可能与差的RFS相关联，而且这种关联与组织学类型无关。而Iwata等^[20]^研究发现p40表达强度不影响患者的远期生存。

p63、p40影响患者生存的可能机制为：基因组的异常特别是染色体区域3q26-3qter的扩增，是肺癌癌变的主要特征。令人感兴趣的是*p63*基因测序谱就是在3q27-29扩增^[25]^。最近有研究^[26]^显示p63和突变型p53在转录激活、诱导细胞凋亡、肿瘤形成、肿瘤的侵袭和转移的过程中相互拮抗。除了细胞凋亡和细胞衰老的调节，p63的缺失也是与各种癌症的侵袭密切相关的因素^[27]^。ΔNp63的一个复杂结构在COOH端，剪接产生外显子导致5种不同的C-末端（α、β、γ、δ和ε）。在特定条件下，ΔNp63α可以通过p53和TA同种型诱导细胞凋亡被下调^[28]^。

查阅文献，未见有关于CK5/6表达与患者预后相关的报道。但有研究^[29]^发现CK5/6联合其它标志物可以预测基底样乳腺癌患者的生存。

## 2 肺腺癌IHC标志物的诊断及预后意义

### 2.1 肺腺癌IHC标志物的诊断价值

#### 2.1.1 TTF-1在诊断肺ADC中的应用

*TTF-1*基因位于染色体14q13，表达产物为38 kDa的核蛋白，位于细胞核，属于Nkx2同源结构域转录因子家族成员。TTF-1特异表达在肺和甲状腺肿瘤。TTF-1蛋白的表达简单，可以直接评估。它完全局限于肿瘤细胞的细胞核，而非细胞质或细胞膜上，不存在反应性非肿瘤基质细胞或炎症细胞浸润。TTF-1可能是目前最好的鉴别肺ADC的标志物，也用于区分肺原发性ADC和来自于甲状腺以外的转移性肺ADC。肺ADC中，TTF-1表达在75%-85% ^[30]^。文献^[31]^报道TTF-1诊断肺ADC的敏感性为60%-89%，特异性为75%-98%。而肺SQCC中TTF-1通常表达阴性，但也有少量的阳性表达的报道，阳性比例在1%-37%之间^[32]^。

#### 2.1.2 Napsin A在诊断肺ADC中的应用

*Napsin A*基因位于染色体19q13.3上，编码的产物为45 kDa含有420个氨基酸的单链蛋白，其表达受TTF-1调控。Napsin A特异表达于肺和肾脏，在肺内表达于肺泡Ⅱ型细胞。Napsin A的IHC染色位于细胞浆。2000年，Hirano等^[33]^首先发现了该标志物主要在肺ADC中的表达。不同的研究中Napsin A在肺ADC中阳性表达的比例约58%-91%^[34]^，需要指出的是低阳性表达率的研究中使用的是组织芯片、活检或细胞学标本。文献^[31]^报道Napsin A鉴别肺ADC的敏感性为83%-90%、特异性为90%-98%，比TTF-1更准确。Napsin A已成为新的肺ADC标志物。研究^[35]^发现与TTF-1相比，Napsin A表现出更强和弥漫的染色。因此在TTF-1弱染色、局灶性染色或结果难以解释的情况下，Napsin A检测特别有用。在几项比较Napsin A和TTF-1表达的研究中，其中一些认为在ADC中Napsin A是一个比TTF-1更敏感的指标^[36]^，但也有相反的结论^[37]^。大多数研究^[7]^表明Napsin A在肺SQCC中不表达，但也有文献^[32]^报道，Napsin A在SQCC中阳性表达率为12.5%-26%。

#### 2.1.3 CK7在诊断肺ADC中的应用

CK7高度表达在气管和尿路上皮细胞。有报道^[38]^认为CK7鉴别肺ADC具有高度敏感性（94%-100%），但特异度不高（53%-78%）。CK7可以帮助识别TTF1和粘蛋白（mucin）染色阴性的肺ADC。CK-7在肺SQCC中也有阳性表达（30%-60%），特别是在周围型SQCC^[36]^。

Mucin也是一个鉴别ADC的IHC指标，但它的敏感性较低（23%-30%）^[39]^。近年来，表面活性蛋白-A（surfactant apoprotein-A, SP-A）和TTF-1，都被确认为是有价值的诊断肺ADC的外周气道上皮细胞标志物。尽管SP-A对肺ADC具有高度特异性，但与TTF-1相比，灵敏度较低。它的缺点是表达直接与组织学亚型和分化程度有关^[40]^。虽然高分化ADC通常强烈表达SP-A，但在诊断最困难的低分化肿瘤中通常不表达。

### 2.2 肺腺癌IHC标志物的预后价值

TTF-1是诊断型肺癌标志物中最早被关注的与生存相关的标志物。但自1999年Puglisi等^[41]^首次提出TTF-1与肺癌患者生存有关后，不断受到质疑。Pelosi等^[42]^研究发现，虽然TTF-1的表达与生存不相关，但有超过75%免疫反应细胞的患者预后更好。在Berghmans等^[19]^的*meta*分析中，有4项研究表明TTF-1阳性和更好的生存有关，5项研究的结果显示TTF-1阳性对生存没有明显影响，甚至有一项研究认为TTF-1阳性是生存不良的预后因素。这些异质性的结果可能是由于以下原因造成：不同的TTF-1阳性评分阈值、使用单克隆抗体浓度不同、染色方法不同和研究的患者群不同。TTF-1表达为何与患者生存相关的原因尚不清楚。但研究^[41]^表明TTF-1可以通过调节细胞增殖与血管形成，促进肿瘤生长。TTF-1的表达与肿瘤的分化程度负相关^[36]^。TTF-1参与肺分化基因的调控，包含SP-A、SP-B、SP-C和10 kDa Clara细胞基因启动子，这一点支持了TTF-1的表达与细胞分化相关^[43]^。此外，有研究^[44]^认为SP-A和Clara细胞基因启动子的表达是早期NSCLC患者生存期延长的一个独立预后指标。最近TTF-1的抑癌作用也被报道，TTF-1下调与肿瘤进展和获得转移能力相关，其原因是在*K-ras*/*p53*突变的情况下，*HMGA2*基因被敲除，解除了阻遏^[45]^。

Napsin A是继TTF-1之后又一个在预后中受到关注的肺ADC诊断型标志物，但有关Napsin A对肺癌患者的预后意义研究较少。Lee等^[35]^研究发现，单因素生存分析显示TTF-1表达阳性预后较好，但无统计学差异；相反，多因素分析显示，无论TNM分期情况怎样，Napsin A是一个比TTF-1更有利的独立预后因素。

目前研究认为Napsin A可影响肺癌的进展。有研究者^[46]^发现致瘤性肾HEK293细胞系中Napsin A表达抑制肿瘤生长。有研究^[34]^观察到分化较好的肺ADC中Napsin A表达超过低分化的ADC。

查阅文献未见到有关CK7直接与肺ADC患者生存有关的报道。但早先有研究^[47]^发现CK7的五个亚型与肺ADC的不良预后明显相关。

## 3 小结和展望

上述标志物诊断肺SQCC和ADC最大的问题是单独应用的敏感性和特异性还不足以进行精确亚分型，通过联合几个IHC标志物作为一个整体进行诊断，可能是今后的一个趋势。虽然以往的研究显示诊断肺SQCC和ADC的IHC指标在评估患者的预后方面有一定的价值，但这些研究结果尚不确定，而且有些结果相互矛盾。因此这些指标与肺SQCC和ADC患者预后的关系，还有待进一步研究。
